# Using single-cell multiple omics approaches to resolve tumor heterogeneity

**DOI:** 10.1186/s40169-017-0177-y

**Published:** 2017-12-28

**Authors:** Michael A. Ortega, Olivier Poirion, Xun Zhu, Sijia Huang, Thomas K. Wolfgruber, Robert Sebra, Lana X. Garmire

**Affiliations:** 1 Cancer Epidemiology Program, University of Hawaii Cancer Center, Honolulu, HI USA; 2 Department of Molecular Biosciences and Bioengineering, Honolulu, HI USA; 3 Icahn Institute and Department of Genetics and Genomic Sciences, Icahn School of Medicine at Mount Sinai, New York, NY USA

**Keywords:** Single-cell sequencing, Cancer, Mutation, Gene expression, Methylation, Heterogeneity, Multi-omics

## Abstract

It has become increasingly clear that both normal and cancer tissues are composed of heterogeneous populations. Genetic variation can be attributed to the downstream effects of inherited mutations, environmental factors, or inaccurately resolved errors in transcription and replication. When lesions occur in regions that confer a proliferative advantage, it can support clonal expansion, subclonal variation, and neoplastic progression. In this manner, the complex heterogeneous microenvironment of a tumour promotes the likelihood of angiogenesis and metastasis. Recent advances in next-generation sequencing and computational biology have utilized single-cell applications to build deep profiles of individual cells that are otherwise masked in bulk profiling. In addition, the development of new techniques for combining single-cell multi-omic strategies is providing a more precise understanding of factors contributing to cellular identity, function, and growth. Continuing advancements in single-cell technology and computational deconvolution of data will be critical for reconstructing patient specific intra-tumour features and developing more personalized cancer treatments.

## Introduction

**Fig. 1:**
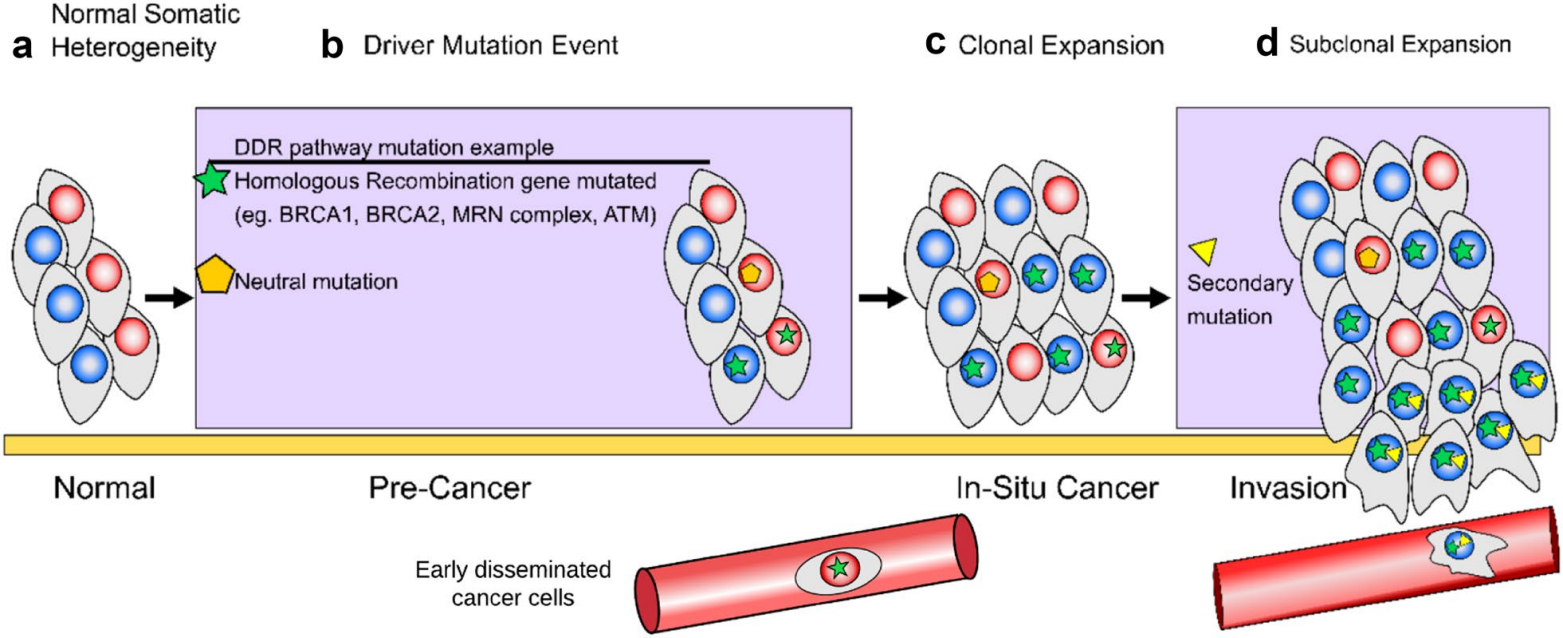
Heterogeneity and metastasis. **a** Normal healthy tissues have a naturally occurring degree of somatic heterogeneity. These mutations can arise due to environmental factors and inaccurately resolved errors in transcription or replication. **b** As mutations stochastically arise, some will be neutral, thus having no apparent affect on the phenotype, while others may occur in ‘driver’ gene regions and have more immediately observable traits. For example, mutated DNA damage response (DDR) genes can drive tumorigenesis because they leave the cell without the necessary pathways to resolve lesions. **c** Driver gene mutations can confer an advantage in the founder clone and promote subsequent expansion. **d** Secondary mutations that occur in subclones further drive heterogeneity and can lead to metastasis. Additionally, recent research suggests that metastases may also derive from early disseminated cancer cells

DNA serves as the source code for specific mechanisms that regulate cellular identity, function, and growth. The genome is generally replicated with high-fidelity. However, stochastic somatic alterations can occur at an average rate of 3 mutations per cell division in normal cells [1, 2]. These genetic changes can be the effect of inherited mutations, environmental factors, or inaccurately resolved errors in transcription or replication. Mutations typically occur in non-coding regions of the genome and have no immediately apparent effect on the phenotype of the cell [2–5]. However, as mutations accumulate over time, they increase genetic variations and the likelihood of developing a neoplasm. Communities of mutations, or alterations to driver genes, can lead to increases in proliferation, a higher frequency of errors in transcription and replication, and/or the enabling of apoptotic evasion [6, 7]. Finally, recent studies indicate that metastases may also derive from early disseminated cancer cells [8]. These features are hallmarks of cancer that subsequently facilitate neoplastic progression (Fig. 1) [9].

To better interpret cellular heterogeneity, researchers have developed various high-throughput applications to generate a more comprehensive cellular atlas of the human body. Tang et al. [10] initially reported a single-cell RNA-seq experiment, where only one cell was sequenced in a single run. This cell was manually separated under the microscope. Since then, the technology has improved several times, each time providing a higher cell count and/or expression sensitivity in a single run. Notably, published in 2012, SMART-seq allowed for greater sensitivity and capturing of full-length transcripts, however cells had to be manually picked in that experiment limiting practical cell capture counts. The Fluidigm C1 capture method introduced microfluidic chips for more automated larger scale cell capture that could be paired with effective library preparation technologies. Starting from 2014, a number of emulsion-based protocols including that by 10× Genomics increased this number by another one to two orders of magnitude (Table 1).

**Table 1:** Notable advancements in single-cell techniques

Year introduced	Notable technology advancements	Method cell range^a^
2009	Tang et al. [10]	1^b^
2011	STRT-seq [23]	< 100
2012	SMART-seq [24]	< 100
2012	CEL-Seq [25]	< 100
2013	Fluidigm C1 (IFC) [26]	< 800
2013	Smart-seq 2 [27]	< 1000
2014	MARS-seq [28]	10,000 s
2015	Drop-seq [29]	10,000 s
2015	inDrop [30]	10,000 s
2016	Chromium (10× Genomics) [31]	10,000 s
2017	ddSeq (Bio-Rad) [32]	10,000 s
2017	SPLiT-seq [33]	10,000 s
2017	Seq-well [34]	10,000 s

This is a non-comprehensive list of peer-reviewed studies that advanced single-cell isolation and preparation techniques

^a^The “range” lists the largest relative population that can or has been studied using this technique

^b^This method involves mechanical separation and isolation of individual blastomeres into single wells

**Fig. 2:**
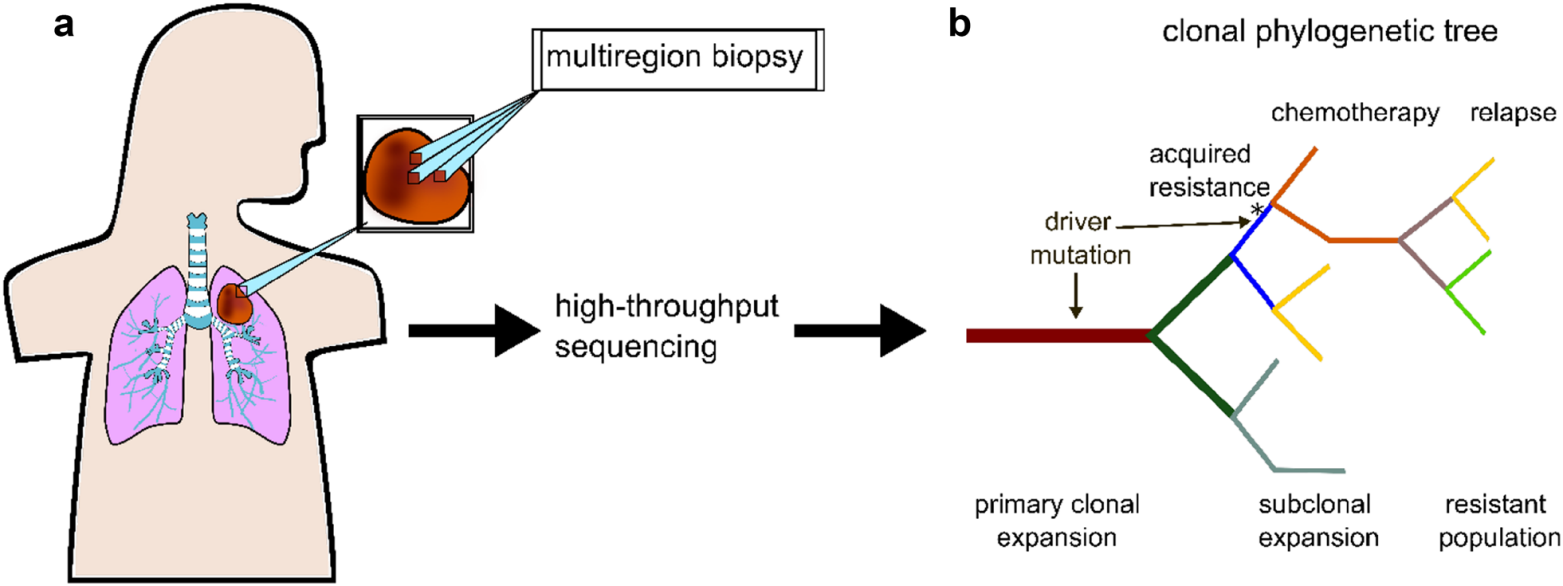
Clonal phylogeny in cancer and resistance. A Darwinian tree model best describes clonal evolution. **a** Multiregion biopsies have been used to investigate intra-tumour heterogeneity. This involves taking biopsies from different regions of the same tumour then preparing high-throughput sequencing libraries. **b** Phylogenetic reconstruction of clonal evolution gives a detailed understanding of heterogeneity in the tumour. A mutation occurring at the ‘trunk’ of the tree and promotes clonal expansion. Subclones arise due to subsequent mutations that diversify the population. Driver mutations can also occur later in clonal evolution and infer resistant properties that were not present in the initial driver mutation. If chemotherapy fails to knock out unique trunks, a drug-resistant population will remain and serve as the dominant feature during relapse

Catching up with the advances in the technology, methods to investigate complex populations are only now coming to fruition with single-cell precision. For example, bulk high-throughput sequencing has been previously used to reveal that intra-tumour genetic and epigenetic heterogeneity progress through sub-clonal branched evolution rather than through linear expansion (Fig. 2) [11, 12]. However, for similar studies, single-cell tools for phylogenetic reconstruction of clonal evolution are more complicated due to lower coverage than bulk samples [13–16]. Characterizing the branched sub-clonal evolution of a neoplasm is critical for identifying key sub-population driver mutations promoting diversification, expansion, invasion, and eventually colonization to other parts of the body. In addition, the aggregated effect of tumour heterogeneity is important to resolve because resistance in one or more clonal subsets of a global tumour cellular population can impact chemotherapeutic efficacy (Fig. 2) [17]. In fact, chemotherapies have a modest overall median survival benefit of 2.1 months while costing around $100,000/year in the U.S. [18, 19]. One option to mitigate this inefficiency is to remodel patient specific intra-tumour heterogeneity computationally using single-cell genomics data and determine functional pathways at a high resolution [20–22]. While the circulating tumor cells provide an opportunity to directly profile the difference when comparing to the primary tumor samples retrospectively, investigating different tumor subgroups allows one to reconstruct the evolution of single tumor cells relative to each other in a more continuous fashion. To assemble a comprehensive cellular map of the body, accurate and reproducible experimental protocols, and computational analysis pipelines will be critical to extract and interpret heterogeneous information. Here, we provide a review of current single-cell genomics strategies developed for investigating cellular heterogeneity.

## Single-cell partitioning

The standard workflow of single-cell investigations includes dissociating a bulk-cell sample into individual cells, isolating those cells, preparing them for the desired application, acquiring data, and analyzing data. Today, methods for separating cells still include mechanical perturbation or enzymatic digestion to separate bulk samples into single cells. However, the downstream methods for isolation, preparation, data generation, and analysis have made rapid advancements. Partitioning bulk samples and isolating individual cells can be technically challenging and necessitates optimization, often on a tissue-specific level. This initial step for investigating heterogeneity through single-cell applications can be complicated by the inefficient separation, which results in higher doublet capture rates in fluidics and droplet-based technologies. Caution should also be taken not to induce unnecessary mechanical or chemical stress on the cell during this process. Performing a clean isolation will also avoid unnecessary molecular debris that can impact the ability to assign individual unique molecular identifiers (UMIs) to single cells during the process of demultiplexing on the cellular and transcript/allele levels.

**Fig. 3:**
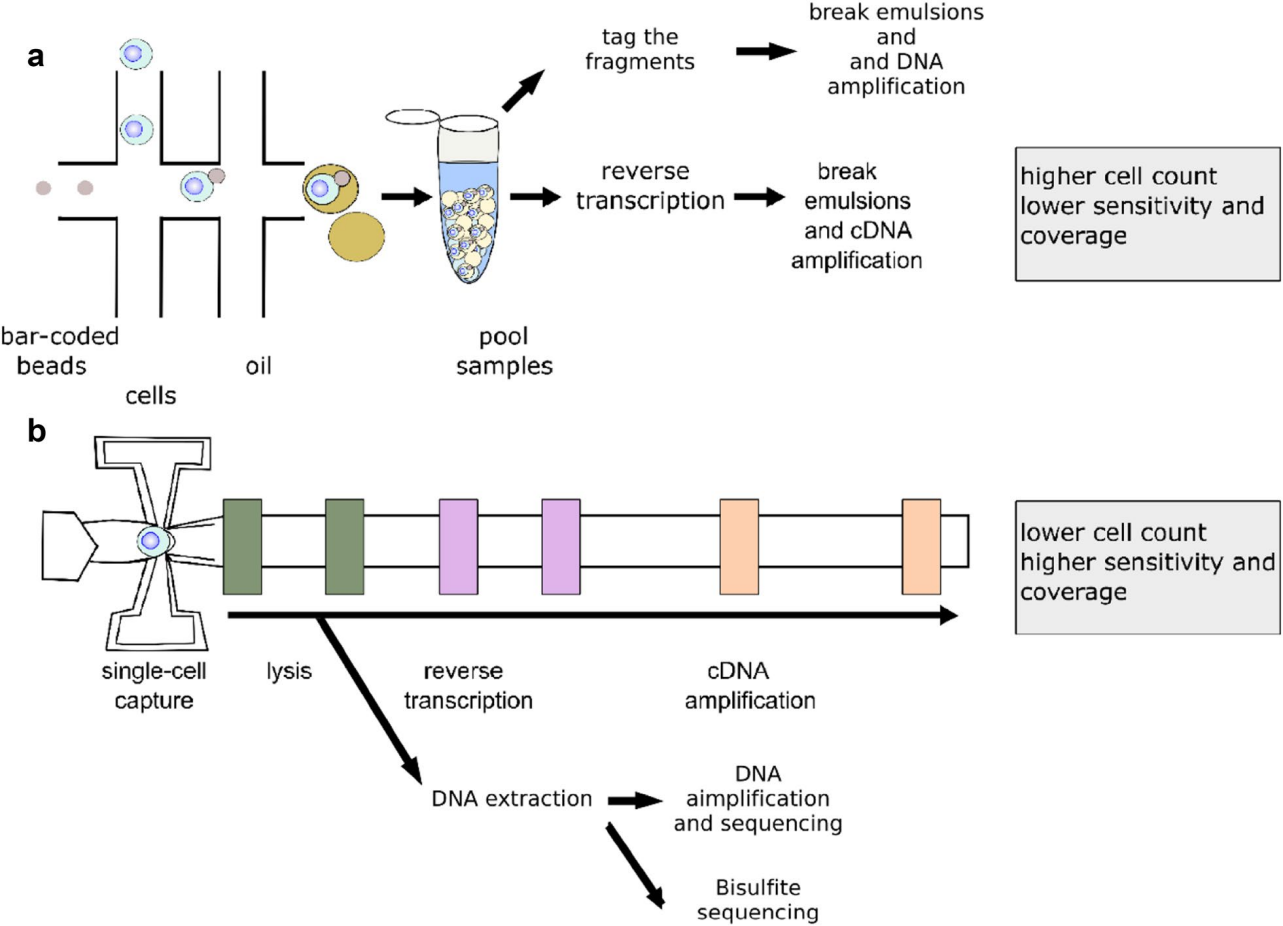
Single-cell isolation and preparation. **a** One method for isolating single-cells is by droplet microfluidics. In the first channel, individual cells are coupled with uniquely bar-coded beads that continue down the pipe until they are captured by an oil droplet. The oil droplets are then pooled in high quantities, and PCR is performed on the population. **b** A second approach for isolating single-cells is to pre-enrich cells by FACS then pass them through an IFC chip, which collects them into individual wells. IFC chips are available in different cell size ranges which assists in limiting the capture more than 1 cell/well. Unlike the droplet approach, PCR is performed on individual cells. This method results in lower overall cell counts than the droplet approach, however, there is a reported trade-off in sensitivity. Overall, droplet-based methods will yield higher numbers of cells per experiment, but the quality of data is more sparse, whereas, microfluidic chip methods provide a deeper cellular profile but with fewer cells. Researchers have to weigh the trade-off of single cell read depth or single-cell population breadth

After separating and suspending the population of tumour cells from biopsy tissue, single cells can either be processed in bulk or sorted and enriched to select specific sub-populations. Most commonly, single cells can be isolated by flow-cytometry, laser capture microdissection, serial dilution, using antibody-coated magnetic beads, or microfluidic-based techniques. Droplet-based technologies such as Drop-seq, inDrop, Chromium, and ddSeq can produce tens to hundreds of thousands of uniquely barcoded cells (Table 1) [28, 35, 36]. The droplet-based approach for isolating and preparing single cells involves using bead-based surface chemistry to facilitate molecular sample preparation methods while encapsulating the cell in an emulsion or aqueous microfluidic partition (Fig. 3a). Each bead contains DNA fragments with unique barcode sequences that are incorporated with cell material during encapsulation. While encapsulated, RNA is also reverse transcribed. The emulsions are then broken prior to pooled amplification and sequencing. Integrated microfluidic circuit (IFC) chips offer an alternative approach to isolate and process cells individually by capturing them in small chambers [37]. IFC protocols have a natural quality-control step whereby doublets can be recorded by microscopy visualization before preparing downstream applications (Fig. 3b).

Costs should be considered when deciding the satisfying number of cells to run relevant to the depth of heterogeneity in the tissue being interrogated. Isolating cell populations through a chip limits the number of cells that can be captured in a single run. It can be more expensive than droplet-based approaches (~ $3.5 versus $0.10 per cell) [38]. In addition to financial differences, these recent approaches and their derivatives each have unique technical concerns and limitations that have been reported in Svensson et al. [39]. After isolating the single-cell population of interest, the subsequent preparation steps vary widely depending on the desired application. Below, we elaborate on exemplary applications for studying single-cell heterogeneity.

## Single-cell whole genome and whole exome sequencing

Fundamental technical concerns exist with generating and analyzing single-cell genetic, epigenetic, and expression data, because of the low amount of starting material. Single cells have ~ 6 pg of genomic DNA, 10–30 pg of total RNA that must be amplified before sequencing, and roughly 250–300 pg of protein that can be analyzed. A battery of computational models have been developed to address false-positives due to nonlinear amplification, false-negative allelic drop-out due to amplification bias, non-uniform coverage, and noise that arises during single-cell genome or transcript amplification (Table 2) [40]. For this reason, bioinformatics and computational biology applications are critical for analyzing NGS output files and accurately identifying genetic variation. Single-cell whole-genome and whole-exome sequencing (scWGS and scWES, respectively) provide amplified genomic DNA variant datasets that can then be used to reconstruct clonal evolution or to measure genetic heterogeneity.

**Table 2:** Tools for investigating heterogeneity

Name	Description	Link	Input	Specific to single-cell	References	Accessibility
Databases						
scRNASeqDB	A database for gene expression profiling in human single cell by RNA-seq	https://bioinfo.uth.edu/scrnaseqdb/	N/A	Yes	Cao et al. [62]	***
The Human Protein Atlas	Spatial distribution of protein expressions	http://www.proteinatlas.org/	Protein name	No	Uhlén et al. [108, 109]	*****
Enrichr	Very complete meta-database	http://amp.pharm.mssm.edu/Enrichr/enrich	List of genes/proteins—BED file	No	Kuleshov et al. [110]	*****
CIViC	Clinical interpretation of cancer variant	https://civic.genome.wustl.edu/home	Gene or variant ID	No	Griffith et al. [111]	****
MyGene2	A portal for sharing health and genetic information	https://www.mygene2.org	Genetic information	No	Xin et al. [112]	****
Genome sequencing						
SCITE	Pseudo-temporal clonal tree construction	https://github.com/cbg-ethz/SCITE	Presence/absence/unknown mutation matrix	Yes	Jhan et al. [113]	*
oncoNEM	Pseudo-temporal clonal tree construction	https://bitbucket.org/edith_ross/onconem/src/76f7122a24c7539fbc29e6589b86110efbf7b13b?at=master	Binary matrix + estimation of FPR and FNR for each SNVS	Yes	Ross and Markowetz [114]	*
BWA	DNA reads aligner	http://bio-bwa.sourceforge.net/bwa.shtml	fastq file + reference genome	No	Li [115]	*
Methylation						
Bismark	Aligner for bisulfite treated sequencing reads	https://www.bioinformatics.babraham.ac.uk/projects/bismark/	fastq files	No	Krueger and Andrews [83]	**
RNA-seq						
Granatum	Graphical pipeline for scRNA-seq analysis	http://garmiregroup.org/granatum/app	Expression matrix and sample metadata	Yes	Zhu et al. [56]	*****
Monocle2	Pseudo-time construction using DDRTree	http://cole-trapnell-lab.github.io/monocle-release/articles/v2.0.0	Expression matrix and sample metadata	Yes	Trapnell et al. [57]	*
scLVM	Subpopulation detection	https://github.com/PMBio/scLVM	Expression matrix	Yes	Buettner et al. [58]	*
PseudoGP	Probabilistic pseudotime for single-cell RNA-seq data	https://github.com/kieranrcampbell/pseudogp	Expression matrix	Yes	Campbell et al. [116]	*
SPADE	Cell hierarchy inference	http://www.nature.com/nprot/journal/v11/n7/full/nprot.2016.066.html	Expression matrix	Yes	Anchang et al. [60]	*
STAR	RNA-seq reads aligner	https://github.com/alexdobin/STAR	Fast	No	Dobin et al. [117]	**
CNV						
InferCNV	Average gene expression on large genomic regions	https://github.com/broadinstitute/inferCNV	Gene expression matrix	Yes	Patel et al. [63]	**
ECdetect	Detection of extrachromosomal DNA	https://github.com/virajbdeshpande/AmpliconArchitect	Bam file	Yes	Turner et al. [118]	*
CNVkit	Detection of CNV from DNA sequencing	https://github.com/etal./cnvkit	BAM file + target regions (BED files)	No	Talevich et al. [119]	**
SynthEx	Detection of copy number alteration and tumour heterogeneity profiling for whole genome and exome sequencing	https://github.com/ChenMengjie/SynthEx	Count data (bed files) + optional vcf files for tumor samples	No	Silva et al. [120]	**
Ginkgo	Web platform for visualization and clustering	http://qb.cshl.edu/ginkgo	Bed files	Yes		*****
MutSigCV 2.0	Eliminate false positive mutations in large datasets	http://archive.broadinstitute.org/cancer/cga/mutsig	Mutations for each sample + sequencing coverage	No	Lawrence et al. [121]	**
HotNet2	Identify mutated subnetworks across pathways and protein complexes	http://compbio.cs.brown.edu/projects/hotnet2/	Mutation data + protein–protein interaction network	No	Leiserson et al. [122]	*
Proteomics						
Wishbone	Reconstructing bifurcating developmental trajectories of single-cells	https://www.c2b2.columbia.edu/danapeerlab/html/wishbone.html	tsv expression files	Yes	Setty et al. [123]	***
Multi-omics						
DeepCpG	Infer missing methylation states and expressive DNA motifs linked to methylation	https://github.com/PMBio/deepcpg	Methylation position file + ref genomes + fastq files	Yes	Angermueller et al. [87]	*
SSrGE	Link gene expression with SNVs. Provide a pipeline to extract SNVs from scRNA-seq	https://github.com/lanagarmire/SSrGE	Expression matrix + binary matrix; fastq files	Yes	Poirion et al. [106]	*
Genetic architecture						
combinatorialHiC	Processing single cell combinatorial indexed Hi-C	https://github.com/VRam142/combinatorialHiC	fastq files + barcodes	Yes	Ramani et al. [124]	*
Others						
Integrate-neo	Gene fusion neoantigen discovering tool	https://github.com/ChrisMaherLab/INTEGRATE-Neo	fastq files (or tsv) + bedpe files + reference genomics	No	Zhang et al. [125]	*
awesome-single-cell	Exhaustive community-driven list of single-cell analytical tools	https://github.com/seandavi/awesome-single-cell	N/A	Yes	N/A	*****

Software, computational packages, and databases mentioned in the paper

The accessibility of a tool is our evaluation of its user-friendliness towards bench scientists who are not necessarily computationally trained

The accessibility ranges from “*” (least accessible) to “*****” (most accessible)

### Experimental methods

To date, three primary techniques are used for whole genome amplification (WGA) which include: (1) PCR-based methods such as multiple annealing and looping-based amplification cycles (MALBAC), (2) degenerate oligonucleotide-primed polymerase chain reaction (DOP-PCR); and (3) non-PCR based method using random hexamers or non-specific priming like multiple displacement amplification (MDA) [41–43]. Each of the three primary techniques becomes unreliable when there is less than 1.5 pg of genomic material. The smaller starting volumes are susceptible to environmental contamination and require optimal sterile working conditions to avoid the creation of false positives [44]. Each method used for single-cell genomic DNA amplification has biases to consider and can be affected by poor coverage or uneven sequencing depth, which will result in noisy and inaccurate read counts.

MALBAC tends to have GC bias, but reports find that MALBAC libraries are more reproducible than those generated with MDA [45]. Some analytic tools, such as the Ginkgo web platform (http://qb.cshl.edu/ginkgo), take measures to reduce or eliminate these amplification biases. According to the authors of Ginkgo, of the three genetic amplification techniques used, MDA has non-uniform coverage and worse GC bias than either DOP-PCR or MALBAC. Reports suggest that despite moderate physical coverage DOP-PCR is the most reliable method [42, 46]. While this Review was being prepared, an approach that uses linear amplification via transposon insertion (LIANTI) was reported that may prove to have less amplification bias and high (97%) genome coverage [47].

### Exemplary applications

With new isolation, amplification, and computational modelling techniques becoming available there has been a general progression toward increased populations of cells or higher quality coverage in rare populations. In 2012, scWGA by MDA of 58 cells was used to demonstrate mutational contributions of SESN2 and NTRK1 in neoplasm progression. In this study, more than 90% of the single-cell genomic data mapped back to the reference genome and they had an 11% allele dropout rate [48]. In another early example, Xu et al. performed scWES-seq of 25 single cells by MDA amplification which was used to reconstruct clonal mutations occurring within PBRM1 and VHL in kidney cancer. Here, the MDA approach yielded a false positive rate of 2.67 × 10^−5^ and an allele dropout rate of 16.43% [49]. A more recent study by Gao et al. used DOP-PCR to amplify genomic content from 1000 cells and reported that the majority of subclonal CNV occurs shortly after the onset of the primary driver mutation in breast cancer. While the false discovery rate was not mentioned in this article, their data suggests that 20–40 single cells were required for detecting subpopulations with 95% power [50]. It is clear that the number of cells being reported in a single study has changed rapidly. However, while the available number of cells per analysis has been used to reach up to nearly 1500 cells (using MDA), the average number of reads per cell decreases as the number of cells increase. Thus, much like scRNA-seq, there is a tradeoff between the individual cell quality and the total number of cells analyzed within a batch [39]. The shortcoming of large batches emphasizes the need for computational tools that can correct such bias (Table 2).

## Single-cell RNA sequencing

The most broadly developed method for single-cell analysis is single-cell RNA sequencing (scRNA-seq). High-throughput RNA-seq of bulk samples from scores of patients have provided novel insights into many cancer types [51–53]. However, deploying RNA-seq analysis at single-cell resolution can provide an even deeper level of understanding the heterogeneous composition of tumour samples by identifying constituents otherwise masked in bulk RNA-seq [54, 55]. The first scRNA-seq was performed in 2009 using single mouse embryo blastomeres [10]. Since then, there has been an increasing surge of sc-RNA-seq publications. Compared to bulk level RNA-seq, scRNA-seq has at least two advantages. First, a more accurate and sensitive presentation of the cell-to-cell variability can be discerned. Second, these data can be reorganized into pseudo-temporal arrangements that can more accurately reconstruct clonal evolution. Beyond experimental advantages, monitoring the dynamics of sub-clonal populations across the course of treatment also has the potential to inform and allow more precise adjustment of therapies.

### Experimental and computational methods

There are several platforms available for isolating and preparing RNA from single cells. One of the greatest technical concerns is in amplifying these low concentrations without introducing significant bias. Data generated by droplet-based approaches permit 10,000 s of cells to be counted, while other platforms that use chip-based systems process only 100 s of cells at a time but are more sensitive to calling the number of genes per cell [39]. After successful isolation of single cells, there are a wide number of molecular approaches to creating scRNA-seq libraries such as SMART-seq, SUPeR-seq, BAT-seq, CEL-seq, and STRT-seq amongst others. The SMART-seq approach can generate full-length cDNA, whereas approaches like STRT-seq (targeting 5′ end of mRNA) and CEL-seq (targeting 3′ end of mRNA) can be used for multiplexing samples [23, 25, 27]. SMART-seq employs a special reverse-transcriptase that anchors both ends of cDNA with distinct nucleotides. The absence of one of these ends eliminates incompletely reverse-transcribed RNA molecules after a subsequent cloning step. It is important to note that sensitivity and accuracy become concerns when the experiments scale up to larger numbers. Here, sensitivity is defined as the smallest quantity of input RNA molecules required for a gene to be confidently called. Accuracy is defined here as the closeness between the estimated and the actual abundance levels of input molecules. Whereas studies have shown that the droplet-based microfluidic approaches permit greater numbers of cells to be counted, chip-based systems appear to be more sensitive [39].

Computationally, many tools have been tailored to take advantage of the high-resolution of scRNA-seq data and deconvolute noise [56]. For example, Monocle2 is an unsupervised algorithm designed to analyze the heterogeneity among cells and reconstruct the micro-evolution timeline from scRNA-seq data [57]. Other tools such as scLVM [58], PseudoGP [59], and SPADE [60] have provided various solutions to analyze heterogeneity with scRNA-seq data computationally. With the scRNA-seq analysis toolbox expanding rapidly, graphical user interface (GUI) pipelines such as Granatum (http://garmiregroup.org/granatum/app) have recently been developed to ensure that accessing the latest development in computational methods is amenable for clinical and non-informatics researchers [61]. In addition, with datasets accumulating at an astonishing speed, there have been efforts like the RIKEN Single-Cell Project (http://singlecell.riken.jp/en/) to consolidate, index, and organize publically available datasets [62].

### Exemplary applications

scRNA-seq has been used broadly to provide data on genetic expression and has now been widely applied to a variety of cancer types. Since there are many more techniques developed and reported for scRNA-seq, we will only highlight a few applications as examples of how scRNA-seq is impacting the discussion on heterogeneity. In one example, the SMART-seq protocol was used to profile full-length mRNA from 430 primary glioblastomas to reveal an intratumor spectrum of differentiation states [63]. The SMART-seq protocol was later improved to increase mRNA yield, coverage, sensitivity, accuracy and reintroduced as Smart-seq 2 [27]. Smart-seq 2 is now a widely used approach in scRNA-seq. In one case, it was used to profile 4347 cells from six oligodendrogliomas and revealed subgroups of undifferentiated cells with a stem-cell-like expression that may be the source of oligodendrogliomas [54]. These data highlight that one benefit of performing expression analysis at single-cell resolution is it can reveal subpopulations otherwise masked in bulk data. In addition, enhancing sensitivity for clonal-level therapies alongside offers the potential for discovering novel, previously undetectable biomarkers on an individual level.

Currently, one of the most interesting shifts in research to recognize is that scRNA-seq is progressing to include a broader range of samples in addition to a deeper pool from a single source. Thus, as the cost of performing scRNA-seq continues to drop, it will facilitate simultaneous inter- and intra-tumour investigations. For example, a recent report using 9879 cells from 10 IDH-A tumours and 4347 cells from six IDH-O tumours were compared with 165 TCGA bulk samples to identify a common progenitor for IDH mutant gliomas [64]. This report is representative of the growing trend to combine available bulk data with single-cell data cohorts for broader and deeper data mining potential. In addition to profiling tissue samples, scRNA-seq is also used to investigate circulating tumour cells (CTC), which is particularly valuable for prospective monitoring [65]. The SMART-seq method was initially reported in an investigation on CTCs and was proposed as a method for identifying candidate cancer biomarkers [24]. Isolating and analyzing expression in CTCs alongside monitoring circulating cell-free DNA (ccfDNA) burden has high direct translational potential for identifying personalized biomarker panels to guide treatment in real-time.

## Single-cell chromosome conformation capture

Developments in studying single-cell genomic architecture have become increasingly deployed to understand the relationship between topology and phenotype. Topology is interesting because single nucleotide variations (SNV), point mutations, and insertions or deletions (indels) can indirectly impact the expression of a distant gene by rearranging the genetic architecture [66]. Since the expression of some genes is affected by long-distance interactors, another level of heterogeneity to consider is the arrangement and proximity of chromosome territories within the nucleus [67]. Genomic architecture is described as being organized in A/B compartments, topologically associated domains (TADs), and loops. Perturbation at any level of these structures can have a significant role in determining domain accessibility which can either improve or inhibit activity in that region. These physical genomic folding structures have been revealed using long-range genomic interaction maps derived from high-throughput sequencing data [68]. When integrated with other techniques such as scWES or single-cell RNA-seq, Hi-C provides an informative tool for identifying the relationship between the 3D architecture of the genome and gene expression [69].

### Experimental methods

To analyze single-cell nuclear DNA structure, high-throughput sequencing is coupled with a high-resolution chromatin conformation capture (3C) assay (sciHi-C) [70]. The chromatin architecture of single cells is reconstructed by generating short and long-range interaction maps. Briefly, interaction is inferred by fixing the DNA, followed by enzymatic restriction digestion, adaptor ligation, and proximity ligation. This sequence of steps allows interacting loops and TADs to be ligated together and will thereby yield a higher number of reads due to more frequent interaction [71]. Pipelines for analyzing multiplexed scHi-C data have recently become open-sourced [71]. On a more local scale, single-cell ATAC-seq (scATAC-seq) is used to profile open (transcription-permissive) chromatin. During scATAC-seq, isolated nuclei are processed by Tn5 tagmentation, which inserts adapters into nucleosome-free regions [72, 73].

### Exemplary applications

scHi-C methods give detailed information about the state of the chromatin accessibility and long-range interactions. These methods are currently being adopted to define how architecture evolves throughout the cell-cycle at single-cell resolution [74]. A recent publication used scHi-C to reveal the architecture during pronuclear formation of G1 zygotes. In the zygote, maternal and paternal pronuclei have different levels of organization with maternal DNA lacking A/B compartments. This suggests that organization of paternal compartmentalization is likely inherited from the sperm [75]. The organization of loops, TADs, and compartments is significant because it infers which regions are more active and perhaps more prone to mutations [7]. On a more localized level, a separate study used a modified sciATAC-SEQ approach called SCI-seq was demonstrated on 16,000 single cells from different cancer types. SCI-seq uses a lithium-assisted nucleosome depletions strategy to remove histones followed by cross-linking than by the scATAC-seq protocol [76]. Together, these techniques provide information on the organization of DNA in the nucleus.

## Single-cell epigenetics

Epigenetic diversity involves heritable changes that affect genomic expression but that do not affect the DNA information. This includes direct modification of nucleic acids (i.e. 5mC, 6mA, m^6^A, and pseudouridine), and post-translational modification of histones (e.g. methylation, and acetylation) [77, 78]. DNA methylation adds another layer of complexity to our understanding of how heterogeneity affects cellular identity and function. Hypermethylation of DNA is associated with transcriptional repression, while the reverse is true for hypomethylation. Single-cell epigenetics studies have advanced more rapidly than those that deal with proteins. Elucidating epigenetic heterogeneity at the single-cell level add a deeper understanding of how methylation patterns are maintained on a clonal level across cell populations and across individuals. However, it should be noted that the most significant roadblock to generating single-cell resolution methylation data is that current techniques are harsh and result in massive loss of DNA template. Also, these techniques often include amplification methods that remove the ability to detect epigenetic modifications.

### Experimental methods and applications

Amongst the experimental approaches inferring cytosine modification, single-cell bisulfite sequencing (scBS) is the most widely used technique. scBS-seq preferentially de-aminates unmethylated cytosine thereby converting unmethylated cytosines to thymines. However, during this bisulfite treatment step, nicks and fragmentation in the DNA occur that reduce the quality and quantity of the input. This is followed by primary and secondary adapter ligation and PCR [79–81]. Single-cell reduced representation bisulfite sequencing (scRRBS) has lower coverage of total CpG sites but higher coverage of CpG islands [82]. Aligned reads generated from this technique require special tools such as Bismark for read mapping and methylation calling [83]. A third technique known as single-cell whole-genome bisulfite sequencing (scWGBS-seq) was developed that does not include the pre-amplification step that takes place in scBS-seq but this approach has lower coverage complexity [84]. Comparatively, the scRRBS method only covers 1% CpG sites across the genome, in contrast to 48.4% of CpG sites by scWGBS. Single-cell methylase assisted bisulfite sequencing (scMAB-seq) and CpG island methylation sequencing for single-cell (scCGI-seq) have also recently been proposed [85, 86]. Future comparative studies of these various methods will assist in determining the differences in mapping and cost efficiency.

Cytosine methylation studies on cancer samples at the single-cell level currently lag behind other -omics approaches. The technical difficulties of bisulfite treatment yield poor coverage, thus these methods might not be able to unveil untargeted features of different cell populations. For this reason, Farlik et al. inferred cell line drug response in developing the scWGBS approach [84]. Finally, recent single-cell studies proposed different analytical tools to correct the input features, such as the use of a deep neural network to reconstruct noisy and missing CpG data [87]. Using computational methods to fill in sparse data will not only rescue poorly resolved data but may also be applied to identify biomarkers, project clonal evolution, or rank potential drug responses.

## Single-cell proteomics

There are many layers to deciphering cell-to-cell heterogeneity. Since there is not a direct 1:1 turnover of mRNA occurrence to protein translation, adopting single-cell proteomic studies provides information on the final layer of inter- and intra-tumour heterogeneity. Unlike genetic or expression analysis, proteomic investigations at the single-cell level lack a way to amplify the starting material. Therefore, single-cell proteomic studies have the technical challenge of developing more sensitive methods for detection.

### Experimental and computational methods

Quantifying proteins at single-cell resolution is a developing technology complicated by the transient nature of functional proteins. Much like other -omics sections covered earlier, investigating the proteome at single-cell resolution requires accounting for low input levels [88]. Single-cell time-of-flight mass cytometry (CyTOF) is one method used to address this issue. CyTOF targets epitopes of interest utilizing antibodies coupled with transient metal element isotopes. Single-cell droplets are introduced to inductively coupled argon plasma where the cell is vaporized, and the atomic constituents are ionized before time-of-flight sampling [89]. Since CyTOF is limited to around 50 parameters, this approach yields a much lower throughput than scRNA-seq. However, it is more affordable than scRNA-seq and can help to determine if an enrichment (e.g. FACS) step is necessary before transcriptome analysis. Another mass spectrometry approach recently proposed is a method known as single-cell proteomics by mass spectrometry (SCoPE-MS). The SCoPE-MS workflow was designed to isolate protein from single-cells and prepare each cell for MS. SCoPE-MS attempts to resolve the issue of protein loss during transfer and low starting material by manually separating and lysing cells. This method includes tandem mass tags for reporter ion relative abundance quantification [90]. Alternatively, non-MS approaches for single-cell proteomic studies can utilize chip-based isolation. In the single-cell barcoded chip (SCBC) method, cells are isolated into wells and probed with antibody arrays which are analyzed by a microarray scanner [91]. Antibody arrays are also utilized in single-cell western blotting (scWB) for which isolated cells are lysed, and SDS-PAGE is applied to each well. Relative to the MS approaches, scWB is limited to probing the sample with a smaller panel of antibodies.

There are a variety of statistical tools available for inferring subpopulations and subpopulation specific markers from single-cell protein data including SPADE, Phenograph, and Wishbone [92]. For example, histoCAT is a new powerful integrative method used to integrate single-cell CyTOF measurement with image-based spatial information to detect spatial and phenotype interaction at the cellular level [93]. Also, new studies propose different methods to construct dynamic protein signalling networks using single-cell protein measurement [94, 95]. In particular, one new approach created a dynamic regulation network from CyTOF measurements to model the drug perturbation of the epithelial-to-mesenchymal transition [96]. This type of approach can then facilitate the discovery of critical events correlated with a cell state transition.

### Exemplary applications

One example of the SCBC method for single-cell chip-based proteomic investigation first heavily dilutes a FACS enriched population before loading them into microchambers. Wei et al. used a phosphoproteomic antibody array to profile mTORki resistant glioblastomas [97]. This SCBC study, cells were lysed after isolation, and the protein contents were captured using custom antibody barcodes. Chip-based methods can assay a relatively larger number of proteins (n > 40) than other techniques currently available, but still fewer the MS approaches [88]. For this reason, the continuing development of single-cell mass spectrometry strategies such as CyTOF will be the technology that unlocks single-cell proteomic scalability. As a recent example, a panel of more than 30 antibodies was used in CyTOF to analyze tumour cells, adjacent normal tissue, and blood from 28 patients with lung adenocarcinoma [98]. The CyTOF data reported here revealed that tumour-infiltrating myeloid cells likely shape the composition of anti-tumour T-cells. Thus, the ability to profile large population of single-cell surface markers has powerful implication in immunology.

## Single-cell multi-omics

Single-cell multi-omic strategies capture the most accurate state of factors that contribute to the cellular phenotype. Ultimately, the integration of several layers of data will be necessary for deconvoluting the relationship between expression, function, and identity. This is because bulk level analysis can only describe the general trends in a population that can mask cellular subtypes [99]. Multi-omic studies are complicated by the technical requirement of separating and preserving different molecular layers from the same cell. Bioinformatics and computational biology provide critical support for reconstructing features that may become noisy as a result of sample loss during multi-omic sample preparation.

### Experimental methods

**Fig. 4:**
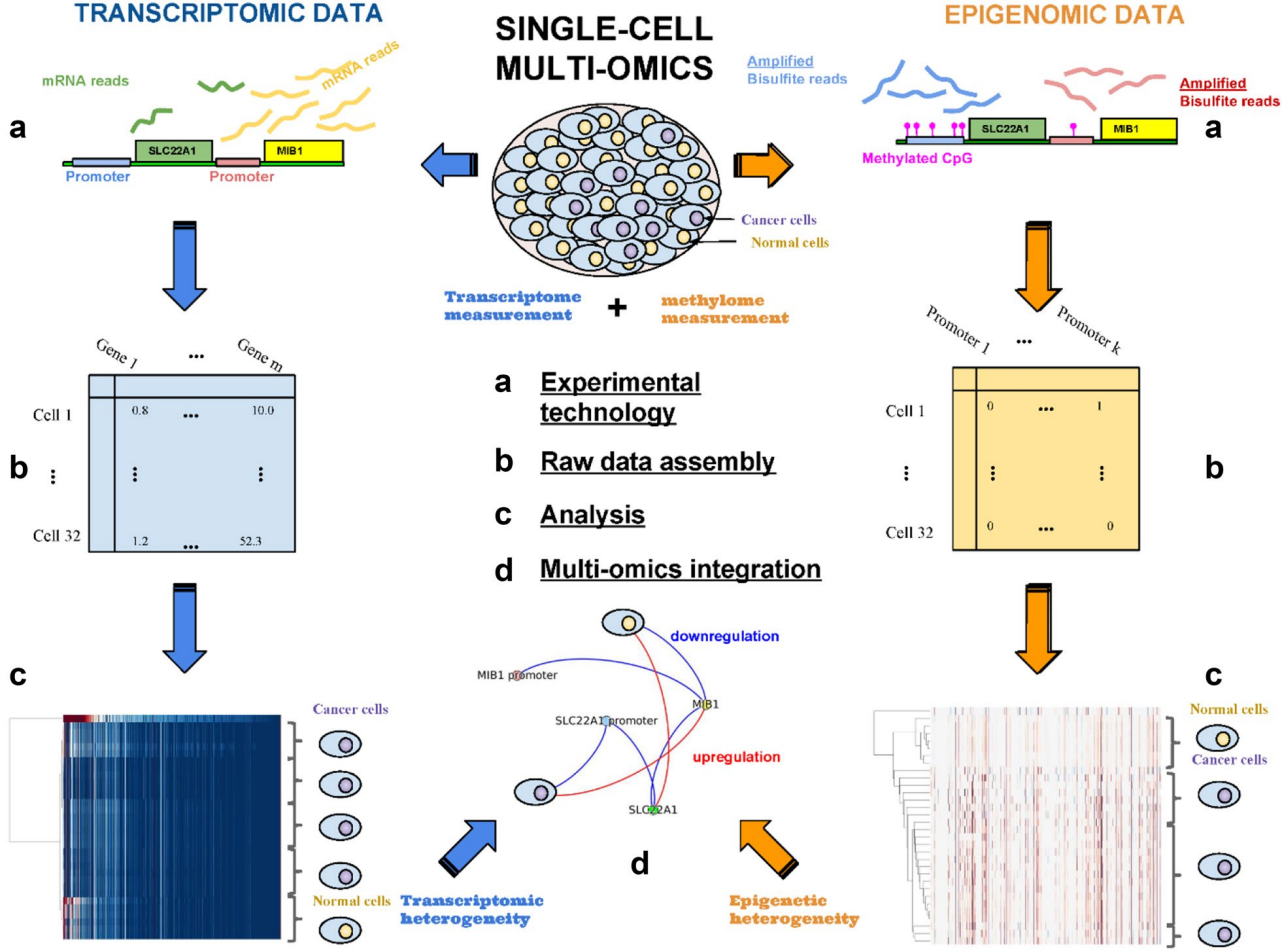
Single-cell multi-omics analysis workflow. **a** Multi-omic technologies can produce reads from the transcriptome (RNA-seq), the genome (exome sequencing), and/or the methylome, from the same cells. **b** Read alignments, quality control (QC), and specific processing steps create “feature expression” matrices, where cells are represented as vectors and genomic features (e.g. gene expression, methylation) represented as columns. **c** The different omic matrices can then be analyzed independently, for detecting cell subpopulations and ranking the genomic features etc. **d** Finally, multi-omics integration can be performed to identify coherent features from different omics that separate different subpopulations

The most cutting-edge research calls for investigators to combine all of the techniques discussed to reconstruct multi–omic single-cell profiles (Fig. 4). Achieving this level of resolution will provide the most comprehensive profile of cell-to-cell diversity in normal and tumour tissue and inform researchers on the impact of single-cell genetic and epigenetic heterogeneity. Bock et al. proposed that molecules collected from the same cell can be assayed by one of several approaches depending on the desired downstream application [100]. For example, two methods described involve either separating the molecular layers (e.g. DNA and RNA) into their equivalents or splitting the sample itself into different fractions and proceeding with the desired isolation within the given fraction. Alternatively, multi-omic methods can be combined into a single workflow [100]. Taken together, the cluster of data generated by multi-omic approaches can infer the underlying triggers of cellular identity and function. As these techniques in cell isolation and amplification continue to improve, multiple layers of heterogeneity reconstruction can be used to identify neoplastic predisposition markers and provide a refined map for precise drug treatment regimen.

### Exemplary applications

Since multi-omic strategies are at the frontier of single-cell research, the body of literature remains relatively nascent. The majority of current available single-cell multi-omics technologies are focused on the link between epigenetic and transcriptional variations. Macaulay et al. established scG&T-seq to simultaneously measure genetic variation and gene expression via separation of gDNA from polyA RNA using a biotinylated oligo-dT primer [101]. scG&T-seq equips Smart-seq 2 for whole transcriptome analysis and offers various methods for DNA amplification depending on the downstream application (MDA, PicoPlex etc.). In contrast to conventional scRNASeq sequencing methods, scG&T-seq utilizes ERCC-spike-ins to assess the number of genes expressed and transcript coverage lengths. Angermueller et al. developed another method called scM&T-seq to evaluate the relationship between methylation and transcription variations in heterogeneous cell populations through scRNASeq and scBS-seq techniques [102]. scM&T-seq was applied in discriminating 61 mouse serum ESCs (embryonic stem cells) and 16 ESCs grown in 2i media. The results showed that the connection strength between methylome and transcriptome varies from cell to cell. Another similar method called scMT-seq applied Smart-seq 2 and scRRBS for single-cell transcriptome sequencing and methylome sequencing, respectively [103]. Compared to scM&T-seq, scMT-seq provides similar CpG islands overlap in a more cost-effective way. This study helped to reveal the relationship of gene expression and DNA methylation in gene body and promoter regions in neuron single cells.

Regarding simultaneous measurement of gene expression with another omics data generated from the same cell, DR-Seq is an example of pioneering work on single-cell DNA and RNA parallel sequencing [104]. Without manually separating the nucleus and cytosolic mRNA, DR-Seq applies a quasi-linear amplification method with predefined adaptors to quantify gDNA and mRNA. Additionally, the comparison of DR-Seq and CEL-Seq showed that the additional steps for amplification of gDNA would not affect the mRNA results. However, this single-pot strategy requires in silico masking of the coding sequences (exonic region) of the genome to determine copy number variation, which leads to incomplete transcripts from the cell. Another recently published work by Stoeckius et al. developed CITE-seq to integrate cellular protein markers and transcriptome in single cells through oligonucleotide-labeled antibodies [105]. CITE-Seq not only enabled to differentiate cellular subgroups based on surface protein expression, but also achieved a consistent output of protein detection with currently standardized flow cytometry. Compared to scRNA-seq alone, CITE-Seq demonstrates both the highly consistent protein and RNA profiles with literature and also an enhancement of characterization of cell phenotypes based on immune cell experiments.

From the analytical perspective, certain additional layers can be reconstructed given one layer is provided. For example, SNVs can be directly extracted from RNA-seq reads and then correlated to gene expression [106]. In addition, by using the input fastq files it is now possible to highlight predictive DNA motifs linked to methylation profiles [87]. However, most of the high-throughput scRNA-seq pipelines are designed specifically for mRNA expression counting. Since the library products are quite short, there is fairly limited capability to do this without modifying more commercial protocols.

Three-omics single-cell assays also became possible. Recently, Hou et al. established scTrio-seq to simultaneously sequence and analyze single cell’s genomic copy number variations, DNA methylation and transcriptomic gene expression together [107]. scTrio-seq demonstrated its ability to efficiently measure DNA methylome, transcriptome and genome copy number compared to scRRBS, bulk cell RNA-seq and bulk cell RRBS and bulk cell WGBS. The integration of triple omics information via scTrio-seq on 25 HCC cancer cells identifies two heterogeneous subpopulations with different malignancy and metastasis potential.

## Conclusions

High-throughput sequencing techniques provide clinicians with a more comprehensive understanding of the genetic and epigenetic heterogeneity in normal and cancer cells. Moreover, future personalized treatments might integrate as a routine single-cell strategy to unveil intra-tumour heterogeneity and thus provide a more accurate therapy regimen. Multi-omics approaches that detail inter- and intra-tumour heterogeneity within individual patients will continue to evolve and provide critical insight to informing more accurate treatment regimens based on prognosticated drug response. In addition, these emerging molecular techniques when combined with computational analysis tools will advance research in other areas such as developmental biology, biotechnology, pathology and more. As larger amounts of single-cell data become publicly available, there will be increased opportunities to identify subclonal-specific biomarkers at a personalized level. User-friendly data portals for single-cell analysis, such as Granatum, will become increasingly integral in the bench-to-bedside transition [61]. The comprehensive annotation and analysis of single-cell datasets will be the foundation of understanding how cell-to-cell variability in normal and cancer cells influence cellular identity and function in the human body.

## Abbreviations

3C: chromosome conformation capture
BAT-seq: barcoded 3′-end specific sequencing
BWA: Burrows-Wheeler Aligner
ccfDNA: circulating cell-free DNA
CD: cluster of differentiation
CEL-Seq: cell expression by linear amplification and sequencing
CIViC: clinical interpretation of variants in cancer
CNV: copy number variation
CNVkit: copy number variation kit
CTC: circulating tumor cells
ddSeq: droplet digital sequencing
DOP-PCR: degenerate oligonucleotide primed PCR
ECdetect: extrachromosomal DNA
EwS: Ewing sarcoma
GATK: genome analysis tool kit
GUI: graphical user interface
IDH: isocitrate dehydrogenase
iEVORA: epigenetic variable outliers for cancer risk prediction analysis
IFC: integrated microfluidic circuit
InferCNV: infer copy number variation
INTEGRATE-Neo: integrate neoantigens
LIANTI: linear amplification via transposon insertion
MALBAC: multiple annealing and looping based amplification cycles
MARS-seq: massively parallel single-cell RNA-sequencing
MDA: multiple displacement amplification
MS: mass spectrometry
mTORki: mTOR kinase inhibitor
MutSigCV 2.0: mutation significance copy variation
NTRK1: neurotrophic receptor tyrosine kinase 1
OncoNEM: oncogenetic nested effects model
scATAC-seq: single-cell assay for transposase accessible chromatin sequencing
scBS: single-cell bisulfite sequencing
scCGI-seq: CpG island methylation sequencing for single-cell
sciHi-C: high-resolution chromatin conformation capture (3C) assay
SCITE: single-cell inference of tumor evolution
scLVM: single-cell latent variable models
scMAB-seq: single-cell methylase assisted bisulfite sequencing
scMT-seq: single-cell methylome and transcriptome sequencing
SCoPE-MS: single-cell ProtEomics by mass spectrometry
scRNA-seq: single-cell RNA sequencing
scRNASeqDB: single-cell RNA sequencing database
scRRBS: single-cell reduced representation bisulfite sequencing
scWES: single-cell whole exome sequencing
scWGBS-seq: single-cell whole genome bisulfite sequencing
scWGS: single-cell whole genome sequencing
SESN2: sestrin 2
SMART-seq: switching mechanism at the 5′ end of the RNA transcript sequencing
SNP: single-nucleotide polymorphism
SPADE: spanning-tree progression analysis of density-normalized events
SPLiT-Seq: split pool ligation-based transcriptome sequencing
SSrGE: sparse SNV inference to reflect gene expression
STRT-Seq: single-cell tagged reverse transcription
SUPeR-seq: single-cell universal poly(A)-independent RNA sequencing
TAD: topologically associated domains
TCGA: The Cancer Genome Atlas
UMI-seq: unique molecular identifier sequencing
WGA: whole genome amplification
